# A framework to assess welfare mix and service provision models in health care and social welfare: case studies of two prominent Italian regions

**DOI:** 10.1186/s12913-015-0800-9

**Published:** 2015-04-09

**Authors:** Francesco Longo, Elisabetta Notarnicola, Stefano Tasselli

**Affiliations:** CERGAS - Health and Social Care Management Research Center, Public Policy Analysis and Management Department,Bocconi University, Via Roentgen 1, 20135 Milan, Italy

**Keywords:** Systems of care, Service provision models, Welfare systems, Health and social care services

## Abstract

**Background:**

The mechanisms through which the relationships among public institutions, private providers and families affect care and service provision systems are puzzling. How can we understand the mechanisms in these contexts? Which elements should we explore to capture the complexity of care provision? The aim of our study is to provide a framework that can help read and reframe these puzzling care provision mechanisms in a welfare mix context.

**Methods:**

First, we develop a theoretical framework for understanding how service provision occurs in care systems that are characterised by a variety of relationships between multiple actors, using an evidence-based approach that looks at both public and private expenditures and the number of users relative to the level of needs coverage and compared with declared values and political rhetoric. Second, we test this framework in two case studies built on data from two prominent Italian regions, Lombardy and Emilia-Romagna. We argue that service provision models depend on the interplay among six conceptual elements: policy values, governance rules, resources, nature of the providers, service standards and eligibility criteria.

**Results:**

Our empirical study shows that beneath the relevant differences in values and political rhetoric between the case studies of the two Italian regions, there is a surprising isomorphism in service standards and the levels of covering the population’s needs.

**Conclusion:**

The suggested framework appears to be effective and feasible; it fosters interdisciplinary approaches and supports policy-making discussions. This study may contribute to deepening knowledge about public care service provision and institutional arrangements, which can be used to promote more effective reforms and may advance future research. Although the framework was tested on the Italian welfare system, it can be used to assess many different systems.

## Background

Beveridge-based systems of health and social care are characterised by the provision of a complex mix of public and private services that are aimed at enhancing the population’s health and well-being. Although these systems were created with the goal of pursuing universal coverage through public service provision, they are dramatically changing because of both financial constraints and societal pressures. The traditional Beveridge-driven configuration of public welfare regimes as being solely responsible for social security has been replaced, particularly in Europe, by institutional networks of various actors with a varied allocation of resources and responsibilities [1,2]. Private and non-profit organisations, together with families, are becoming increasingly more involved in planning and providing health care and social welfare services [3]. Determining how the relationships between these categories of actors affect service provision and progressively shape the nature of welfare systems is a major challenge for both managerial and policy research (cf. Esping-Andersen [4,5]). In this light, the current paper aims to develop a theoretical framework for understanding how service financing and provision occur in health and social care systems that are characterised by a variety of relationships between different actors. We then test the suggested framework on two cases of social welfare and community health care in two prominent Italian regions, Lombardy and Emilia-Romagna. Building on both theoretical and empirical insights, we suggest new directions for welfare policy and management. We focus on the broad policy field of health care and social welfare service provision because it represents one of the most critical issues faced by aging European societies and thus may be representative of the overall welfare system [6,7]. This policy field traditionally features a high level of interaction between public, private, and non-profit organisations and families for both service financing and provision, seeking to address problems such as poverty, elderly-related issues, caring for people with disabilities, child protection, homelessness, mental disease, and substance abuse.

The regions selected for our case study, Lombardy and Emilia-Romagna, have 10 million and 4.4 million inhabitants, respectively, which represents 24% of the Italian population and approximately 30% of Italy’s gross income product (500 billion euros). These two regions have consolidated and mature welfare systems that have experienced national social reforms since the early 2000s. Politically embedded in a liberal-Catholic culture, Lombardy’s welfare system is strongly based, at least in its inspiration, on a purchaser-provider split that is achieved by contracting out services to private providers and on users’ free choice between service providers. The social-democratic political background of Emilia-Romagna led to a more inclusive welfare system that has a strong focus on public planning and community work. We use the case study method to verify the validity of an emerging theory proposed by Eisenhardt [8] and Eisenhardt and Graebner [9]. These two case studies provide evidence for the efficacy and feasibility of the proposed theoretical framework in assessing welfare systems.

### Welfare mixes: Which mix can move the paradigm forward?

The welfare state has long been a research subject, and its intrinsic complexity has been addressed by a number of disciplines that have adopted a range of perspectives [10]. In addition to the rich literature on different welfare systems and distinct models of health and social care provision [3,11], investigations of the crisis faced by the traditional welfare models have increased in intensity in recent decades [12,13]. In Europe in particular, comparative welfare state research has focused on three main branches of models and changes: categorisation, retrenchment, and convergence [14].

Although different conclusions have been reached by different branches of research on the modelling and crisis of welfare, there is general agreement that theory and research have shifted from investigating welfare states to investigating welfare systems [15], adding to the traditional analysis of public institutions that have been historically devoted to welfare the analysis of other public and private actors with the idea of the community as a whole. The natural consequence of this evolution is that service management in the health and social service sector is no longer limited to public-sector dominance but involves a wide network of actors that includes families and private, public, and non-profit organisations for both service financing and provision [16,17].

This issue has been debated in both social policy analysis (since Esping-Andersen [4]) and social administration and public management, where it has also been addressed as “dualisation” [18] or “dualisation of social services” [19], although neither a unique definition nor a systematic interpretation has yet been provided.

Studying welfare systems is therefore a very challenging task. Comprehensive approaches are so rare that many attempts may be similar to “describing an elephant touching only its hide, its foot or its trunk” ([10], p. 153).

The first step in solving this puzzle requires identifying theoretical categories to provide more conceptual clarity. What is meant by “welfare mix,” with its specific focus on health and social care services? More to the point, what are the key issues that need to be analysed to identify the peculiarities and explain the variance between different health and social care systems? If a well-considered definition of welfare mix is “the combined, interdependent way in which welfare is produced and allocated between state, market, family and the third sector” [3], we should explore how to investigate these interdependences and on which issues we should focus our analysis.

Building from the previous literature on welfare mix in the field of health and social care provision, we distinguished between different theoretical approaches that were summarised from the literature. Each of these approaches moves from different items of analysis to highlight welfare mix features and identify a set of items that define welfare systems in modern times. The choices of different variables and the ways they are put together produce differences between the three conceptualisations that we synthesised as 1) welfare provision; 2) regulation and governance; and 3) norms, values and relations (for a synthesis of these approaches, see Table 1).

**Table 1:** **Theoretical approaches to welfare mix: definitions and key variables**

**Approach**	**Authors**	**Welfare mix definition and features**	**Variables used to investigate welfare mix**
Welfare provision	Esping Andersen 1999 [23]; Vogel 1999 [20]; Powell and Barrientos 2004 [22]; Dahlberg 2005 [21].	Welfare mix concept is applied only to the supply and production of welfare services:	“*Nature and geography of providers”*
		- Mixed market theories;	
		- Different industrial models and features of services delivery forms are used to describe welfare regimes;	
		- Reasons leading private actors to enter some public markets are also investigated.	
Regulation and Governance	Goodin and Rein 2001 [32]; Ascoli e Ranci 2003 [25]; Barr 2004 [22]; Bode 2006 [33]; Theobald 2012 [26].	Welfare mix is defined as a network in which welfare is providing by combining funding, production, delivery and regulation of supply and demand. In addition, institutional frameworks and governance procedures are considered.	“*Nature and geography of providers”*
			“*Governance rules*” *“Resources”*
			“*Services features and standards”*
Norms, values and relationships	Evers and Laville 2004 [27]; Zimmer 2000 [28]; Borgonovi 2002 [29]; Pavolini 2003 [30].	Welfare mix is defined as moving from norms and values that generate welfare participation. It is also investigated considering relationships and interactions among different actors as well as decision-making processes and participation mechanisms.	“*Policy definition and values”*

#### Welfare provision

In the welfare provision approach, the concept of welfare mix is studied only with regard to the supply and production of welfare services in the context of mixed-market theories. Therefore, welfare is analysed from a market or quasi-market perspective that considers only the provision of in-kind services. Different industrial models and features of these forms of service delivery are used to describe welfare regimes, and reasons that push private actors to enter some public markets are also investigated [20,21]. Here, social risk coverage is associated with production [22]. Esping-Andersen [23] categorised welfare regimes based on how they pool and cover social risks. The key variable that is used is thus the “*nature and geography of providers*,” meaning analysing different production models, the characteristics of single providers, and market access systems and regulating the public sector.

#### Regulation and governance

Under the regulation and governance approach, welfare mix networks are viewed from a wider perspective by adding other dimensions to the definition of welfare provision. This evolution may be exemplified by Barr [24], who defines welfare provision as the combination of funding, production, delivery, and regulation of supply and demand. Not only the presence of different actors in a welfare economy but also institutional frameworks and governance are considered [25,26]. Welfare is analysed through a broader perspective, extending beyond providing in-kind services to include policy making and system financing. Multiple variables are used in this case: “*Nature and geography of providers*,” as in the previous approach, as well a “*Governance rules*,” “*Resources*,” and “*Service features and standards*.” Governance issues address the allocation of functions (such as regulation, planning, and monitoring) between public and private actors. Resource issues focus on analysing the mix of public and private resources but also on the systems’ financing mechanisms. Service issues bring together the perspectives of users and citizens to explore the coverage of their needs and the peculiarities of guaranteed benefits.

#### Norms, values, and relationships

In the third research stream, scholars analyse the circumstances that induce public and private actors to participate in the welfare mix by examining the norms and values that generate welfare participation [27]. Other authors focus on the relationships between welfare actors [28-30]. In addition, decision processes and participation mechanisms are considered relevant. The principal variable used here is “*Policy definition and values*”, where policies are defined as the different policy-making styles that lead to policy processes and where values represent the ideological backgrounds and norms that inspire welfare.

These existing contributions help advance the debate and theory on welfare service provision and widen the traditional research focus. Both families and non-profit, private and public organisations are given more room for action. Moreover, it is increasingly recognised that decisions are made not at the central level but by a plurality of actors at the local level [31].

At the same time, a unique and clear definition in the literature of what is included in the welfare mix approach is missing, and there is a lack of identification of the variables that determine a mix. A welfare mix is often considered an abstract concept, and it is not always clear whether it is defined as a determinant or a result of welfare systems. Some studies conclude that the mix of different actors is derived from welfare characteristics, whereas others conclude that the mix influences welfare outputs and determines its peculiarities [22,32]. In addition, welfare mix studies may be victims of rhetoric and value judgments concerning the roles of families, the third sector, and communities in general. Finally, the qualitative methods that are used in the welfare literature that uses qualitative approaches are often supported by simultaneous and integrated quantitative analysis [13,14].

Despite the importance of the welfare mix approach and the relevance for modern welfare studies of studies based on this approach, the existing literature provides fragmented contributions that in turn are subject to the risks of providing only generic representations of welfare systems. To guarantee the robustness of this research stream and to realise the potential of the welfare mix approach, this theoretical gap must be filled with clearly identified variables that can define the policy field borders and capture the main features of welfare mixes with an effective methodological framework to analyse modern welfare systems, which is what we provide in the following paragraphs. The assumptions that we derived from the extant literature are summarised in previous paragraphs: welfare mix is the best theoretical model for representing the structure of modern welfare systems; the most significant variables that define welfare mixes relate to institutional arrangements and governance (allocation of functions and responsibilities and decision-making processes) and in particular, regulation, provision, financing, resource allocation and inspiration of welfare systems; managing welfare service provision means focusing not only on production models but also on service features and user needs. An analysis based on these premises should match qualitative information with evidence-based data. For this reason, our suggested framework adds a set of data to complete the qualitative description that can help represent emerging welfare characteristics rather than declared ones for an in-depth analysis.

### A framework for comparing and assessing welfare systems

The analytical framework we suggest and test in this paper is built on the strengths of existing contributions and seeks to fill their information gaps and design a comprehensive approach for comparing and assessing health and social care systems. The aim of the framework is to design a systematic checklist to analyse and compare welfare systems that strikes a balance among completeness, depth, and research feasibility in terms of effort and possible results. We examine national and regional systems and refer to welfare by considering its social and community health care components. Social and community health care has been recognised and acknowledged as representative of welfare regimes because of this type of care’s intrinsic relevance and increasing importance, especially in aging societies [6]. In discussing social and community health care, we include all personal services and benefits (cash or in-kind) that are targeted for inclusion and protection, and we exclude services related to acute health care, pension, or education systems.

Building on the theoretical models discussed in the literature on welfare mixes, we provide a set of variables aimed at capturing and interpreting the different models. From the welfare provision perspective [20,21], we include variables that describe service-provision modes and their funding mixes (formal provider features, informal caregiving features, and relevance). We consider all regulation and governance approach items [25,32,33], but we adopt a wider perspective to analyse system financing, resource allocation, service planning, and public-purchasing modes. We use norms and values [27] to analyse the welfare perimeter and examine targeted individual and social rights. We include formal and informal welfare resources and services and attempt to capture the overall interaction between actors [29] to assess possible synergies or gaps. Finally, we add two missing perspectives: (1) service features and related access mechanisms and the levels of needs coverage and (2) the explicit criteria or implicit mechanisms for users’ selection. We are interested in service features, in particular in the way services are provided, and we focus on quality and service standards because they may explain the costs and welfare intensity per user. This approach is closely related to eligibility criteria and needs coverage.

To summarise, these elements led us to produce a comprehensive framework that comprises six sets of items: (1) a formal policy field definition and its ideological background and values; (2) institutional governance; (3) the public and private resources involved; (4) the nature and geography of providers; (5) the types of service features, standards (service intensity per user), and provision; and (6) users’ access mechanisms, implicit and *de facto* selection criteria, and needs coverage.

To apply this framework, an evidence-based approach is suggested to obtain integrated and parallel quantitative and qualitative data and to produce more valuable policy proposals [34,35]. Our analysis aims to capture both emergent substantial facts and declared formal mechanisms: for this reason, it relies on empirical data and observations and formal documents and statements. Any gap between declared rules or policy programs and emerging welfare characteristics will be considered part of the framework results. The interpretation of data and evidence is more robust if a comparative and longitudinal perspective is possible because it sheds light on the similarities and divergences.

The research methodology suggested here therefore relies on a mixed approach: a qualitative analysis of formal declared policies, governance structures, rules, values and quantitative data about resources, service standards and costs, provider features, selection criteria, and needs coverage, which capture the emergent policies.

We test the efficacy and feasibility of this framework by comparing two Italian regional social and health community care systems that are historically based on opposing declared ideologies.

### Comparison between the welfare systems of the Lombardy and Emilia-Romagna regions

We chose Emilia-Romagna and Lombardy not only because they are two of the most important Italian regions because of their sizes but also because they were among the first to implement comprehensive social care reforms. Moreover, they are representative of two polar welfare systems because of their sociopolitical and rhetorical assumptions. Lombardy has been driven by a liberal-Catholic cultural approach towards enhancing focus on public–private–non-profit collaborative competition in the provision of health care and social welfare services and a leading emphasis on citizens’ freedom of choice in selecting services providers. Emilia-Romagna has been driven by a post-social-democrat approach, with an emphasis on the role of public actors as key players in both analysing and discovering relevant citizens’ needs and providing publicly funded services.

It may be useful to emphasise the current settings in the Italian social care sector that drove our decision to explore regional rather than national systems. With the approval of Law 328/2000 [36], regions were delegated to implement welfare policies; as a result: “*regional and local governments have followed different pathways in the implementation of the reform, in some cases even contradicting the guidelines of the national legislator*” [37]. Today, regions are responsible for their governance structures, in particular for managing the public budget devoted to health and social care and for governing the network of public, private, and non-profit actors who provide care to citizens.

## Methods

We used our proposed framework to build and assess two case studies. We collected data from multiple sources and mixed qualitative research with evidence-based, quantitative data to achieve completeness and to provide triangulation for our findings [38]. Data collection was conducted in five separate steps in 2012, which took approximately eight months. The data were collected specifically for this research because all of the data are unpublished, and they were provided and elaborated by participating institutions (the Emilia-Romagna and Lombardy health and social welfare regional directorates) in adherence with public data protection guidelines. Specifically, we followed the ethical regulations defined by the “Decreto Legislativo 231/2001”, an Italian national law that regulates the access to and use of public data by public administrations, hospitals, and other public institutions.

Data collection and analysis consisted of four separate steps. First, we collected data through in-depth interviews with a research panel composed of social care managers from the regional directorates for both Lombardy and Emilia-Romagna. During these interviews, we asked the managers about the specific regional documents that contained data or information that could be relevant for our analysis. Subsequently, the managers sent us the official documents regarding social and community health care policies in the two regions in the last year (from 2000 to 2012), including legislative and normative documents, official regional reports, regional strategic plans, regional accounts, and budgeting documents. These documents helped us to collect information concerning formal rules, values, governance mechanisms, and official data about financial expenditures and numbers of users served.

Second, we defined a list of detailed indicators that could help our analysis (including data about the mixture of financial resources available, the numbers of users, the average cost of social care intervention per user, and provider characteristics). All of the data were already available from the public organisations (municipalities and local health authorities [LHAs]). The strong commitment of the regional governments allowed for an optimal return rate of the requested data at both the regional and local levels: this allowed us to improve and better understand the aggregate regional information and to integrate the information with local and analytical perspectives.

Third, we used the epidemiological and statistical prevalence of dependency to assess the potential needs for different social targets and then determine the levels of needs coverage granted by the two welfare systems.

Fourth, the data and evidence were discussed in two focus groups (one for Lombardy and one for Emilia-Romagna). The participants were chosen from among the most important social care managers and civil servants at both the regional and local levels. The focus groups were conducted similarly to half-day research seminars; the case studies were presented and the results validated and discussed by regional key players. The participants were managers and practitioners from the regional level, and they all provided us with consent statements for both the focus groups and the interviews. Considering that no patients were enrolled in the focus group and no sensible information was discussed during them, no ethical statement was required because none was necessary. By focus group, in this paper, we mean participant observation of a meeting of professionals and managers who shared perceptions, opinions, beliefs, and attitudes towards their work activity. For this purpose, we obtained the consensus of all of the individual professionals and managers who participated in the focus group and of their home institutions, following the “Decreto legislative 231/2001“cited above.

Next, a number of individual interviews were conducted in each region with single key players to better understand the social care systems (with a focus on the distance between formal and emerging characteristics) and deepen the analysis of certain critical issues that arose within the focus groups.

The data regarding Emilia-Romagna refer to 2012, whereas the data concerning Lombardy refer to 2011. When complete regional data were not available, we relied on data on Bologna and Milan, the most important metropolitan areas in the two regions. We also provide a special focus on long-term care (LTC) for the elderly and disabled given the importance of these interventions for the aging European population.

In the next paragraphs, we present the Lombardy and Emilia-Romagna case studies through the lens of the six variables we defined in our framework.

## Results

### Policy field definition and values

Emilia-Romagna and Lombardy have very different welfare values that come from two traditions of opposing political settings: characterised by a strong liberal environment, Lombardy is more family and market oriented, whereas Emilia-Romagna has a social-democrat background and is more community oriented, with an emphasis on public planning.

Lombardy places a focus on public and private partnership in service provision and individuals’ options to select their specific service mixes. In contrast, Emilia-Romagna is characterised by an emphasis on considering public actors to be responsible for planning and satisfying citizens’ needs, with an ancillary role played by private actors.

Emilia-Romagna’s social care system may be characterised as having four core values [39]: (a) centrality of the local community: individuals, institutions, families and non-profit organisations are responsible for implementing social policy; (b) prevention and social promotion rather than compensation for social risks; (c) personal autonomy and independent living are the main welfare goals; (d) integration between different policy fields (social, health, labour, gender, and education). Public actors are supposed to collaborate with rather than delegate to private actors and are perceived as directors of the system. Families are recognised as sites of social relations and caring and must be sustained.

In Lombardy [40], the social care system was defined as a network of family-centred interventions and policies that provided choices from among different commercial providers and that featured horizontal and vertical subsidiarity together with quasi-markets. Public actors act as regulators, whereas private actors (for-profit or non-profit) provide services in a quasi-market regime. This approach is based on the notion that a welfare society is superior to public institutions alone.

In recent years, both systems have introduced changes in their orientations: Emilia-Romagna has reinforced the goal of integrating social and community health policies, and Lombardy has introduced elements of integration between different social actors and made explicit the need for forms of pooling between public and family resources.

### Governance rules

Local municipalities in Emilia-Romagna play a strong role in both health and social care, with a general push towards decentralisation at the local level. The result is a strong interaction between the social care and health care components. Social care is embedded in municipalities, whereas health care is provided by LHAs, which are branches of the national health care system. General strategies and guidelines are defined at the regional level. At the local level, the most important actors are the social and community health care districts, which administer both social and community health care. Resource administration is managed by LHAs, which balance the stronger policy role of municipalities. The system is thus highly decentralised and based on cooperation between public social and community health programs and providers, which are often non-profit organisations.

In Lombardy, the Regional Directorate is responsible for defining the policies, strategies, and guidelines for social care interventions together with community health care programs. The local system is directly steered by the regional government and has a silo-based structure. The local authorities that are responsible for social care are the social districts, administrative aggregations of local governments with coordination and management tasks. Community health care programs are steered by LHAs, which are actually an operational arm of the regional government with weak coordination with social care.

Financing is provided by a combination of different sources: national financial resources (specific funds dedicated to welfare interventions such as the National Fund for Social Policies); regional resources; and local resources (municipalities’ budgets). Today, national funds are highly irrelevant given the important reductions made in 2010 [41] that made them residual resources.

Resource allocation mechanisms differ based on the different flows of resources. In Emilia-Romagna, for example, the regional social fund is transferred to local municipal authorities with per-capita criteria adjusted by redistribution mechanisms that consider demographic and social factors (i.e., number of resident immigrants; number of children; dependency ratio), whereas the regional LTC fund is transmitted on a per-capita basis to the LHAs, which manage it.

In Lombardy, the social fund is allocated to the social districts following a combination of historical expenditure criteria and per-capita criteria. This fund is allocated first to the LHAs, who then transmit it to the social districts and also have an ex ante and ex post monitoring role. The health care component of community health interventions is transmitted to LHAs and managed directly by them without any form of coordination with municipalities and social districts.

### Resources

The two systems are similar in terms of the public budget allocated to welfare interventions, with the total per-capita social care expenditures being 1.125 euro in Lombardy and 1.121 euro in Emilia-Romagna. These values are the sums of the different cash and in-kind interventions that are provided to families from the different public actors who are part of the social and community care systems. In both regions, families receive, directly from the National Social Insurance Institute, many cash allowances that can be used to finance informal care and other form of assistance related to dependency and disability, with a significant per-capita distribution, near 735 euro in Lombardy and 720 euro in Emilia-Romagna (data publicly available from the website of the National Social Insurance Institute). Other interventions are promoted from the regional and local levels. In Lombardy in 2011, 390 euro per capita were made available by these actors: 169 euro from the region, 123 euro from the municipalities, 5 euro from the province, and 93 euro paid directly by the citizens through copayments for in-kind services. In Emilia-Romagna, the per-capita expenditure on social care in 2012 amounted to 407 euro: 151 euro from the region, 187 euro from the municipalities, 9 euro from the province, and 54 euro paid directly by the citizens through copayments for in-kind services.

This finding is noteworthy because the breakdown of resources (money governed by public actors vs. money provided by citizens through private resources or national cash allowances) shows similar patterns in the two regions (see Table 2). In Lombardy, 74% of overall resources are made directly available to citizens (828 euro, of which 89% is offered through disability allowances and the remaining through out-of-pocket expenditures for copayment), and only 28% are controlled by public actors (40% by municipalities, 59% by the region, and only 1% by the province). In Emilia-Romagna, 69% of resources are controlled directly by users (774 euro, of which 85% is provided through disability allowances and the remainder through out-of-pocket expenditures for copayment) and only 31% by public institutions (50% by municipalities, 40% by the region, and 10% by the province). This evidence is particularly relevant if it is interpreted with respect to policy definition and values: although common wisdom and the dominant rhetoric in the Italian debate on the welfare system posit that Lombardy prioritises citizens’ welfare expenditure copayments while Emilia-Romagna prioritises the public provision of welfare services to citizens, the two systems are impressively similar in their resource mix allocations.

**Table 2:** **Resources: per capita expenditures in the two regions**

**Mix of public and private resources – euro per capita**	***Emilia-Romagna***	***Percentage***	***Lombardy***	***Percentage***
***Regional funds***	€ 151	13,47%	€ 169	15,02%
***Municipal funds***	€ 187	16,68%	€ 123	10,93%
***Province funds***	€ 9	0,80%	€ 5	0,44%
***National cash allowances to families***	€ 720	64,23%	€ 735	65,33%
***Out-of-pocket expenditures***	€ 54	4,82%	€ 93	8,27%
***Total***	€ 1.121	100,00%	€ 1.125	100,00%

### Nature and geography of providers

With regard to the nature of the providers, and focusing on the specific case of structures for LTC and disability, in Lombardy 50,124 of the overall 62,249 existing beds are owned and managed by private providers (80%). A total of 95% of these beds are accredited by the region (i.e., they meet defined quality requirements and deliver services within a public scheme), and thus, they are financed with regional and local funds for an average of 44% of the monthly cost per patient (on average, the public revenues are 1,500 euro per patient per month), whereas the other 56% are co-funded by patients themselves through copayment fees (1,650 euro per patient).

Quite surprisingly, with reference to the premises presented in the policy field definition and values paragraph, private providers play a significant role in Emilia-Romagna. In the province of Bologna, which is the most populous and important province in Emilia-Romagna, 51% of the existing beds in LTC structures are not contracted to any public scheme and thus are entirely financed by users, for an average required monthly family contribution of nearly 2,300 euro. The remaining 49% of beds are accredited and contracted by public programs, which require users to pay a fee amounting to approximately 45% of the monthly service costs. This evidence stands in contrast to the values declared by the two regional governments.

In both regions, public and private providers are generally characterised by small sizes and hyper-fragmentation, mainly in dependency and disability residential care and day care.

Here, we must highlight a typical Italian phenomenon, namely, the existence of an important grey market of informal caregivers for dependent elderly. Our evaluations reveal that there are between 28,000 and 32,000 informal caregivers in the Milan area (where the number of estimated dependent elderly is approximately 40,000), with private expenditures of 320 million euro per year, and 23,000 informal caregivers in the Bologna area (where the number of estimated dependent elderly is 42,926), with private expenditures of 280 million euro per year (see Tables 3 and 4).

**Table 3:** **Nature and geography of providers: financing mix**

**Service financing mix**	***Emilia-Romagna***	***Lombardy***
***Services managed by private providers***	51%	80%
***Providers who receive public funding***	49%	95%
***Cost per patients covered by public funding***	55%	44%

**Table 4:** **Nature and geography of providers: Informal care**

**Informal care**	***Emilia-Romagnav***	***Lombardy***
	***Bologna area***	***Milan area***
***Number of informal caregivers***	23.000	28.000–32.000
***Families’ out-of-pocket expenditures***	280.000.000 euro per year	320.000.0000 euro per year

### Service features and standards

With regard to service provision and standard definitions, a number of elements may be considered (Table 5). In Emilia-Romagna, social districts retain the purchasing function for community interventions that are financed with public resources. Providers may be contracted by municipalities for social services and by LHAs. Public production is frequent, and it is often realised by public social service companies that are active in many social care fields in both the residential and home care sectors.

**Table 5:** **Service features and standards**

	***Emilia-Romagna***	***Lombardy***
***Public actor in charge of service purchasing***	Social districts	Social Districts for issues related to social care.
		Local Health authorities for issues related to community care
***Service provision***	Public production is frequent	Contracting out is frequent
***Regional Government % of expenditure for dependent elderly***	53%	57%
***Out of pocket expenditure of families for nursing homes***	23.000–32.000 euro per year	20.800 euro per year

Similarly, in Lombardy, social districts are responsible for purchasing social care services, whereas LHAs contract for community health services. Users (or citizens) may be free to choose between different alternative providers from among those that have been contracted by local authorities. Contracting out to private for-profit or non-profit producers is preferred. Concerning the regional intervention mix, we again found similarities. In both regions, the expenditure is primarily dedicated to dependent elderly (57% in Lombardy and 53% in Emilia-Romagna), then to disabled adults (31% and 35%) and families and children (6% and 10%).

In Lombardy, the average cost per user differs substantially across regional areas: in the disability day care field, for example, the annual cost incurred by families and public actors may vary between 7,000 and 18,000 euro with an average regional cost of 8,500 euro. For nursing homes for the elderly, the annual cost incurred by families is 20,800 euro on average (out-of-pocket expenditure without considering public contribution). In Emilia-Romagna, the annual cost of nursing care for families varies from 23,000 to 32,000 euro for non-contracted structures (51% of the total) and 18,000 euro per year for public contracted structures (49% of the total) without considering public contributions.

### Eligibility criteria and needs coverage

Eligibility criteria in both Emilia-Romagna and Lombardy vary not only between different care fields but also between different regional areas. Different social districts may use different eligibility criteria, based, for example, on personal income, family income, health conditions, or employment status. If we consider the number of dependent elderly living in the two regions (which we have estimated using the national statistical prevalence index of dependency), we discover that there are approximately 40,000 dependent elderly in the Milan area and 43,000 in the Bologna area. If we consider elderly who receive some type of service from the public social care system, we find that this number corresponds to 25% of the potential dependent elderly in Milan and 26% in Bologna. Making the same estimation for disabled adults living in these two areas, we found that only 20% in Milan and 28% in Bologna receive any type of services from the public system.

Another major issue concerns the mechanism of service provision in terms of the mixture of home, day, and institutional care services. Lombardy’s system emphasises institutionalising patients who need LTC, with more than 62,000 beds available (for 9.8 million inhabitants). Home care is less prevalent, serving fewer than 15% of LTC patients. Emilia-Romagna shows a similar endowment of structures dedicated to institutionalisation, offering 36,000 beds (for 4.4 million inhabitants). Home care services are more prevalent in this region and cover on average 28% of the need expressed by elderly patients. The broad need that is unmet by publicly funded services is primarily covered by informal care, provided by either relatives or professional caregivers, with very similar patterns found in both regions.

Overall, the above analysis of the fragmentation in financing and expenditures reveals similar conditions in Lombardy’s and Emilia-Romagna’s care systems (see Table 6). Most of the coordination activity that integrates the service networks is conducted by families, who must combine different types of public and private expenditures to organise assistance for relatives who need care.

**Table 6:** **Coverage of needs**

	***Emilia-Romagna***	***Lombardy***
	***Bologna area***	***Milan area***
***Number of resident dependent elderly***	Approximately 43.000	Approximately 40.000
***Coverage of elderly (age > 65) dependents’ needs***	26%	25%
***Informal professional caregivers***	23.000	30.000
***Coverage of disabled adults (age 18–65) with needs***	28%	20%

## Discussion

The proposed framework helps us to compare the health and social care systems in two prominent Italian regions: Lombardy and Emilia-Romagna. These two regions have highly different declared welfare values. Lombardy has traditionally determined an overall accountability mechanism based on consumer choice and related financial incentives to selected competitive providers. Meanwhile, in Emilia-Romagna, there is a firm belief in the community and in public planning to guide public interventions towards creating partnerships.

Because of these differences in values, heterogeneous governance structures have been implemented. Lombardy has a more centralised system, whereas there is more room for local governments in Emilia-Romagna. Lombardy features a classic “silo”-based organisational structure that separates social care and that is operated by inter-municipal networks from community health care and managed by LHAs. The community-based approach used in Emilia-Romagna leaves room for local government initiatives and the promotion of social capital. Public planning is crucial, and some room is also left for public providers. Integration between community health and social care policies and budget management is regarded as a means to fostering local government networks and cooperation within communities.

Different values and governance arrangements are not related to significant differences in resource allocation for public welfare programs, and nor are they related to differences in needs coverage. Ultimately, these different systems invest the same amount of public resources, define comparable priorities, and register similar outcomes. The collected evidence about the gaps between declared values and policy rhetoric and the emergent system features, is relevant to the proposed framework because the findings suggest the importance of studying and comparing public discourses and emergent policies to be able to discuss their correlations.

We found that the suggested welfare assessment framework used in this exercise was comprehensive and efficient in capturing all of the relevant issues while at the same time also establishing clear borders for the investigated policy field to better understand its internal correlations and dynamics. Combining values with governance structures, providers and service features, per inhabitant and per user expenditures, and the needs coverage levels helps to build a complete view of welfare policies while remaining sufficiently efficient and feasible for researchers or policy analysts to be put into action.

Assessing welfare systems is broad and complex. There is always the risk of being too general or losing sight of the details. With the proposed framework, analysts and readers obtain a general overview of welfare policies along with some insights into crucial issues such as needs coverage, the opportunity to consider different causal correlations, and the ability to explore specific areas in depth while retaining the opportunity to return quickly to the general picture. To conclude, in the following paragraph, we discuss the main pillars of our proposed framework and their implications for policy analysis and design.

### A tool box to assess welfare system: policy recommendations and managerial issues

In synthesis, the two case studies show that significant elements of local welfare systems can be efficiently available for policy makers and researchers who are interested in social and community care if the proposed dimensions are used as an interpretative framework.

Each of the proposed dimensions alone is useful for shedding light on a specific issue, but crossed interpretations are also significant. Specifically:

1. The ***policy field*** dimension is needed to clearly assess the boundaries of the welfare mission and, in particular, which individual, citizen, and community rights are protected. Moreover, this variable is needed to clearly assess the system’s inspiring theoretical assumptions and therefore its declared orientation: Is the welfare system oriented towards specific target groups or is it universalist? Is it centred on community, family, or individuals? Having a clear understanding of the system’s assumptions and declared premises can help to interpret the other welfare features and to find the gap between formal and emerging welfare arrangements.
2. The ***institutional governance of the policy field*** dimension analyses the role of each public actor within various governance arrangements. By comparing the formal roles assigned to public actors with the funding and provision mixes, we may better understand the emergent institutional settings. Previous research has explained that institutional governance regulates four different items [22]: who pools resources and how; how resources are allocated and redistributed in the welfare system; who plans and controls the structural providers and the facility landscape and how; who purchases and controls providers’ services or distributes cash to users and how.
3. ***Resource mix*** entails a mix of public and private financial resources and informal resources. This approach helps researchers and policy makers to understand the true importance of each actor involved. Specifically, we consider all types of resources that are dedicated by welfare systems to meet individual or community rights: public and private expenditures and financial and nonfinancial resources. Examining the total amount of resources available in a policy field helps in understanding the overall potential of welfare interventions and the mix between public and private expenditures, which can be compared with the declared policy values.
4. The ***nature of service providers*** dimension investigates public and private provider features: are they for-profit or non-profit, formal or informal, concentrated or fragmented? In addition, it relates to the financing mix of private production to understand the actual role of public and private actors. Formal providers may be large and concentrated or small and fragmented. They may have rich and complete service portfolios that autonomously offer integrated service bundles, or they may be very specialised and focused on limited care phases or processes within a disintegrated service landscape. They may be public, non-profit, or for-profit organisations. They may have different revenue mixes funded by public agencies or private expenditures. Informal providers may be relatives, friends, or even individual caregivers paid by users. All of these features can influences care arrangements and thus are strategic issues to be monitored by policy makers.
5. The ***service features*** dimension analyses both institutional settings with regard to service provision and the realised mixture of interventions. This variable is useful for understanding how policies are implemented and which types of services are then provided to citizens. Services may differ in their quality standards and intervention intensity. Support to users may be realised through cash or in-kind services. Cash benefits may be used autonomously without any oversight, or they may be discussed or reported to a supporting agency. Services differ in their contents and settings (home, day facilities, institutions), and they may be offered on an individual basis or through user groups.
6. With the ***coverage of needs*** dimension, we seek to shed light on the effectiveness of social care systems in meeting social needs, defined using epidemiological baselines rather than solely formalised demands. We take for granted the assumption that social care eligibility criteria may differ. They may be explicit and allow a broad range of potential users to select only the appropriate gate or program, or they may be implicit and slightly opaque, which reduces the number of potential users who can make such a selection. Coverage of needs, measured as the percentage of potential users who benefit from public interventions, is a measure of welfare inclusiveness.

Combining the perspectives on declared values and official governance structures with a focus on expenditures, service features, and needs coverage helps to reveal both alignments and unexpected misalignments between regional systems and between their declared values and emerging strategies. In our exercise, two regional governments that have always been considered opposites in terms of their values, institutional arrangements and results reveal similarities when their expenditures, resource allocations, and outcomes are explored.

Using both case study research methods to investigate these regions’ values and institutional arrangements with hard evidence regarding cost per user, resource allocation, and needs coverage, among other areas, allows us to better understand the dynamics of the two regional systems. Under our approach, the case-study-based interpretation of welfare policies is grounded in facts and figures, and the data are selected and processed in light of a strong research hypothesis based on declared programs and values to be verified [6]. In this light, the proposed welfare assessment tool may incentivise interdisciplinary collaboration between welfare experts with different backgrounds because they all converged into a common analytical framework.

The framework is sufficiently flexible to allow for its use in a very deep and analytic approach, when there is an abundance of time and resources to perform the assessment, but it may also be executed using a simplified version by running only a few indicators for each of the six suggested perspectives. This framework can be applied at different levels of government (national, regional, local) and can be used to compare two or many cases in a static or, even better, longitudinal perspective. Collected data may be easily used to promote focus groups and discussion panels between welfare actors, scholars, and policy makers.

## Conclusion

The aim of this paper was to empirically test the heuristic efficacy of the proposed framework that was used to analyse the complex relationships between public and private actors in a mature welfare mix setting. Through a theoretical framework and two case studies (the Lombardy and Emilia-Romagna regions in Italy), we showed that a double perspective is needed in these sorts of analyses: a qualitative perspective to investigate governance characteristics and typologies of services delivered to citizens and a quantitative, evidence-based perspective to examine the provided descriptions and underline the possible differences between the planned interventions, service geography, and needs coverage.

We demonstrated that public service provision depends not only on declared values and formalised policy programs but also on the interaction between different actors, the allocation of governance functions (e.g., regulations, financing, or service access), provider features and the implicitly emergent perimeter of welfare.

The suggested assessment framework appears to be efficient and feasible; it fosters interdisciplinary approaches and supports policy-making discussions. This study may contribute to building a deeper understanding of public service provision and institutional arrangements to promote more effective reforms. The opportunity to apply this assessment framework to many other different welfare systems may advance future research.

## References

[1] Heitzmann K (2006). A new welfare Mix? Theory and evidence for the policy field of poverty relief.

[2] Chatterjee P, Miller D, Chatterjee MA (2013). Visions of Health Policy: A Comparative Case Study of Seven Modern Countries. Soc Dev Issues.

[3] Powell M, Barrientos A (2011). An audit of the welfare modelling business. Soc Pol Admin.

[4] Esping-Andersen G (1990). The Three Worlds of Welfare capitalism.

[5] Bouckaert G, Peters BG, Verhoest K (2010). The coordination of public sector organizations: Shifting patterns of public management.

[6] Daly M, Lewis J (2000). The concept of social care and the analysis of contemporary Welfare States. Br J Sociol.

[7] Jensen C (2008). Worlds of welfare services and transfers. J Eur Soc Pol.

[8] Eisenhardt K (1989). Building Theories from Case Study Research. Acad Manag Rev.

[9] Eisenhardt K, Graebner M (2007). Theory Building from Cases: Opportunities and Challenges. Acad Manag Rev.

[10] Øverbye E, Castles FG, Leibfried S, Lewis J, Obinger H, Pierson C (2012). Disciplinary Perspectives. The Oxford Handbook Of The Welfare State.

[11] Abrahamson P (1999). The Welfare Modelling Business. Soc Pol Admin.

[12] Powell M, Hewitt M (2002). Welfare State And Welfare Change.

[13] Castles FG (2004). The Future Of The Welfare State. Crisis Myths And Crisis Realities.

[14] Schubert K, Hegelich S, Bazant U, Schubert K, Hegelich S, Bazant U (2009). Current State Of Research And Some Theoretical Considerations. The Handbook Of European Welfare Systems.

[15] Heitzmann K (2010). Poverty Relief In A Mixed Economy.

[16] Hughes OE (2012). Public management and administration.

[17] Anheier HK (2014). Nonprofit organizations: Theory, management, policy.

[18] Seeleib-Kaiser M (2011). Welfare State Transformations. Comparative Perspectives.

[19] Bode I (2010). Social Care going Market. Institutional And Cultural Change Regarding Services For The Elderly. J Comp Soc Work.

[20] Vogel J (1999). The European Welfare Mix. Institutional configuration and distributive outcome in Sweden and the European Union: a longitudinal and comparative perspective. Social Indicators Research.

[21] Dahlberg L (2005). Interaction between voluntary and statutory social service provision in Sweden: a matter of Welfare Pluralism, Substitution or Complementarity?. Soc Pol Admin.

[22] Powell M, Barrientos A (2004). Welfare Regimes And The Welfare Mix. Eur J Pol Res.

[23] Esping-Andersen G (1999). Social Foundations of Post Industrial Economies.

[24] Barr N (2004). Economics Of The Welfare State.

[25] Ascoli U, Ranci C (2003). Il Welfare Mix in Europa.

[26] Theobald H (2012). Combining Welfare Mix And New Public Management: The Case Of Long Term Care Insurance In Germany. Int J Soc Work.

[27] Evers A, Laville J (2004). The third sector in Europe.

[28] Zimmer A, Zimmer A (2000). Welfare Pluralism and health care: The case of Germany. The third sector in Germany.

[29] Borgonovi E, Vittadini G (2002). Che cos’è il welfare Mix?. Liberi di scegliere. Dal Welfare State alla Welfare Society.

[30] Pavolini E (2003). Le nuove politiche sociali. I sistemi di Welfare fra istituzioni e società civile.

[31] Kasza GJ (2002). The Illusion Of Welfare Regimes. J Soc Policy.

[32] Goodin R, Rein M (2001). Regimes on pillars: alternative Welfare State logics and dynamics. Publ Admin.

[33] Bode I (2006). Disorganized welfare Mixes: voluntary agencies and new governance regimes in Western Europe. J Eur Soc Pol.

[34] Head BW (2008). Three Lenses of Evidence-Based Policy. Aust J Publ Admin.

[35] Hansen HF, Rieper O (2010). The Politics of Evidence-based Policy-making: The Case of Denmark. Germany Pol Stud.

[36] Legge n. 328 dell’ 8 Novembre 2000: Legge quadro per la realizzazione del sistema integrato di interventi e servizi sociali.

[37] Montanelli R, Turrini A (2008). Evaluating the Reform of Social Services in Italy: a Comparative Analysis. Int J Publ Admin.

[38] Bryman A, Bryman A (2008). Mixed methods research: combining quantitative and qualitative research. Social research methods.

[39] Legge Regionale Regione Emilia Romagna n. 2 del 12 Marzo 2003: Norme per la promozione della cittadinanza sociale e per la realizzazione del sistema integrato di interventi e servizi sociali.

[40] Legge Regionale Regione Lombardia n.3 del 12 Marzo 2008: Governo della rete e degli interventi e dei servizi alla persona in ambito sociale e sociosanitario.

[41] Marano A (2011). I tagli all’assistenza in Italia. Motivazioni e conseguenze. Rivista italiana delle politiche sociali.

